# Quantitative proteomic analysis and replacement therapy identifies haptoglobin as a therapeutic target in a murine model of SLE-associated diffuse alveolar hemorrhage

**DOI:** 10.3389/fvets.2024.1431738

**Published:** 2024-08-12

**Authors:** Ninghui Yan, Chenyi Shao, Yan Zhen, Xueliang Zhang, Nana Xia, Qiang Guo

**Affiliations:** ^1^Department of Rheumatology, Renji Hospital, Shanghai Jiao Tong University School of Medicine, Shanghai, China; ^2^Ren Ji Hospital, Jiading Branch, Shanghai Jiao Tong University School of Medicine, Shanghai, China

**Keywords:** diffuse alveolar hemorrhage, systemic lupus erythematosus, proteomics, haptoglobin, differentially expressed proteins

## Abstract

**Background:**

Diffuse alveolar hemorrhage (DAH) is a catastrophic clinical syndrome and one of the manifestations of pulmonary involvement in systemic lupus erythematosus (SLE), which is characterized by hemoptysis, diffuse pulmonary infiltrates, and respiratory failure. However, the treatment options for DAH remain limited, and DAH-related studies are needed to explore more effective therapeutic directions for better disease management and improved prognosis.

**Methods:**

This study utilized the pristane-induced DAH murine model to mimic the pathological process of DAH in patients with SLE. Proteomic analysis was conducted to detect differentially expressed proteins (DEPs) in the plasma of surviving and non-surviving mice, followed by an analysis of biological functions and pathways. The most significant DEP was then confirmed in the plasma of SLE patients with or without DAH and DAH murine model with or without fatal outcomes. Finally, the therapeutic value of haptoglobin (Hp) replacement was validated in a DAH murine model through lung histopathology, RT-qPCR, and survival analysis.

**Results:**

This study identified 178 DEPs, with 118 upregulated and 60 downregulated DEPs in the non-survival group. Within a set of notable Kyoto Encyclopedia of Genes and Genomes (KEGG) pathways, complement and coagulation cascades emerged as the most prominent pathway associated with the process of DAH. Later, the most significant DEP, haptoglobin (Hp), was confirmed to exhibit a significant decrease in the plasma of individuals with SLE-DAH and DAH murine model with poor outcomes by the ELISA test. Finally, compared with the control group, the severity of DAH in the Hp treatment group was alleviated significantly, as manifested by the decreased levels of pro-inflammatory cytokines (IL-6 and TNF-α), increased levels of anti-inflammatory cytokines (IL-10 and TGF-β), and decreased mortality.

**Conclusion:**

A reduction in plasma Hp levels was observed in SLE-DAH, and the replacement therapy with Hp could alleviate pulmonary hemorrhage and reduce mortality in DAH mice. This study identified Hp as a potential biomarker for its clinical diagnosis and a direction for treatment.

## Introduction

Diffuse alveolar hemorrhage (DAH) is a catastrophic clinical syndrome and one of the manifestations of pulmonary involvement in systemic lupus erythematosus (SLE) (1). Although the incidence of DAH in SLE is low, its mortality rate is as high as 68–75% (2). Patients with DAH typically present with symptoms such as dyspnea, cough, fever, and occasionally hemoptysis (1, 3). Laboratory findings, including anemia and decreasing hemoglobin values, serve as markers of intrapulmonary blood loss (3). The disease can progress rapidly within a few hours or days, evolving from asymptomatic imaging abnormalities to severe life-threatening respiratory failure (1). The mechanism underlying diffuse pulmonary hemorrhage in SLE patients remains unclear. However, the main pathological manifestations of DAH include antibody-mediated pulmonary capillary inflammation, mild alveolar hemorrhage, and diffuse alveolar injury (4–6). Particularly in the early stages of the disease, damaged epithelial or endothelial cells release pro-inflammatory factors and chemokines, leading to the aggregation of inflammatory cells, which, in turn, exacerbate lung damage (7, 8). In clinical practice, the treatment options for this catastrophic complication are quite limited. In addition to basic therapies for DAH and systemic autoimmune diseases, such as high-dose glucocorticoids and cyclophosphamide (9, 10), the primary approach relies on supportive care. This includes hemodynamic correction, blood transfusions, and respiratory support, which are essential measures for managing acute visible hemorrhaging (11). Limited studies have reported that appropriate antibiotic therapy and umbilical cord germination stem cell transplantation could improve the outcomes (12, 13). Therefore, exploring more effective therapeutic strategies for DAH is of great clinical significance for disease management and improving prognosis.

Pristane (2, 6, 10, and 14-tetramethylpentadecane, TMPD) is an isoprenoid alkane used to induce a lupus-like autoimmune syndrome in non-autoimmune-prone strains of mice (14). C57BL/6 (B6) mice generally exhibit alveolar hemorrhage within 2 weeks after injecting pristane, characterized by alveolar and perivascular inflammation, endothelial damage, and hemorrhage, which closely resembles the pathological process of DAH (15–17). In recent years, proteomics has emerged as an innovative frontier in identifying key disease-causing molecules and therapeutic targets. While genomics defines potential gene products, proteomics provides a more accurate reflection of the actual phenotype. However, no relevant proteomic study has delved into the pristane-induced DAH murine model. Therefore, we conducted an in-depth proteomic analysis of this model, aiming to uncover meaningful proteins and potential mechanisms and provide further insights into the treatment of DAH.

Haptoglobin (Hp), an acute plasma phase glycoprotein, is known for its high affinity to bind with free hemoglobin (Hb) in plasma, playing a pivotal role in tissue protection and prevention of oxidative damage (18). It is important to highlight that in the context of diseases associated with hemolysis, such as sickle cell anemia, sepsis, transfusion reactions, and subarachnoid hemorrhage, hemoglobin itself acts as a critical pathogenic factor, and these conditions often exhibit haptoglobin depletion and respond well to haptoglobin supplementation (19–22). It has been reported that Hp intervention can promote the clearance of exogenous hemoglobin in the lung of the murine model and alleviate lung injury and inflammation, which may be related to the iron mobilization pathway (23).

In this study, we conducted plasma proteomic profiling of the pristane-induced DAH murine model encompassing both non-survivors and survivors. To the best of our knowledge, this is the first study of its kind. Based on data-independent acquisition (DIA) methods, the proteomic analysis was performed to identify differentially expressed proteins (DEPs), which were subsequently subjected to the KEGG pathway and GO analysis. Hp, the most significantly downregulated DEP found, was subsequently validated through interventions in animal models and confirmed in patients with DAH.

## Results

### Histological characteristics of the lung tissue in the DAH model

**Figure 1:**
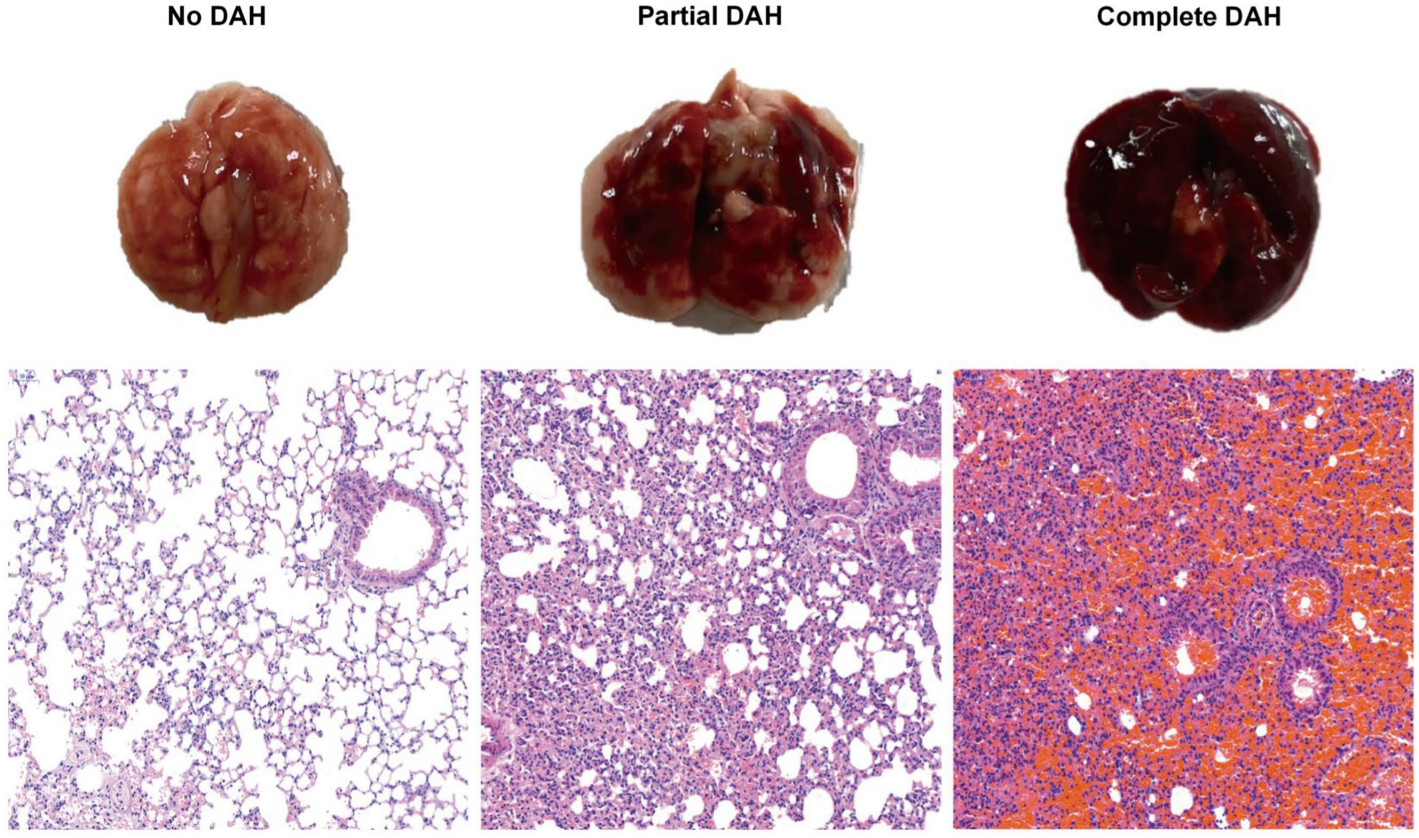
Histological analysis of DAH lung tissue after treatment with pristane. Incidence of DAH was assessed by gross pathology; representative H&E staining of lung sections (200x) from pristane-treated C57BL/6 mice without DAH, with partial DAH, and with complete DAH from left to right.

Intraperitoneal injection of pristane induces diffuse alveolar hemorrhage (DAH) in C57BL/6 mice with lupus. Within 2 weeks, these pristane-treated mice develop severe DAH. On the 14th day, examination of lung tissue sections of pristane-induced mice reveals bleeding under different conditions. Based on the infiltration degree of immune cells and red blood cells, the mice were categorized into three grades: No DAH, Partial DAH, and Complete DAH (Figure 1).

### Establishment of the DAH model

We induced the DAH model in 30 C57BL/6 mice with a single dose of pristane and conducted a 60-day health status observation. Venous blood was collected on days 7, 14, 28, 42, and 60. By day 60, we categorized the mice into two groups based on survival outcomes: 19 mice were in the survival group, and 11 were in the non-survival group. The remaining mice were harvested, and the samples were systematically collected for further analysis. Later, DIA analysis was performed on the peripheral blood collected on the 14th day from both the survival and non-survival groups (Supplementary Figure S1).

### General information on DIA analysis results

To comprehend the pathogenesis of DAH, we conducted a proteomic analysis to compare the differential protein expression between survival and non-survival mice. The polypeptide lengths ranged mainly from 7 to 28 amino acids, with a predominant length of 10 amino acids (Supplementary Figure S2A). Most protein molecular weights were between 10 and 100 kDa, with the majority being 10–20 kDa (Supplementary Figure S2B). Subsequently, we evaluated the distribution of protein coverage and found that a significant proportion of identified proteins exhibited substantial peptide coverage: 39.46% showed over 10% of peptide coverage and 17.66% showed over 20% of peptide coverage (Supplementary Figure S2C). Notably, there was a negative correlation between molecular weight and protein sequence coverage. Principal component analysis (PCA) assessed the relationship between the survival and non-survival groups (Supplementary Figure S2D). There were five biological replicates in the survival group and six biological replicates in the non-survival group. Our findings suggest that a subset of the identified proteins may be responsible for the differences between these two groups in the DAH murine model. Further analysis of the data was conducted subsequently.

### Functional analysis of differentially expressed proteins

**Figure 2:**
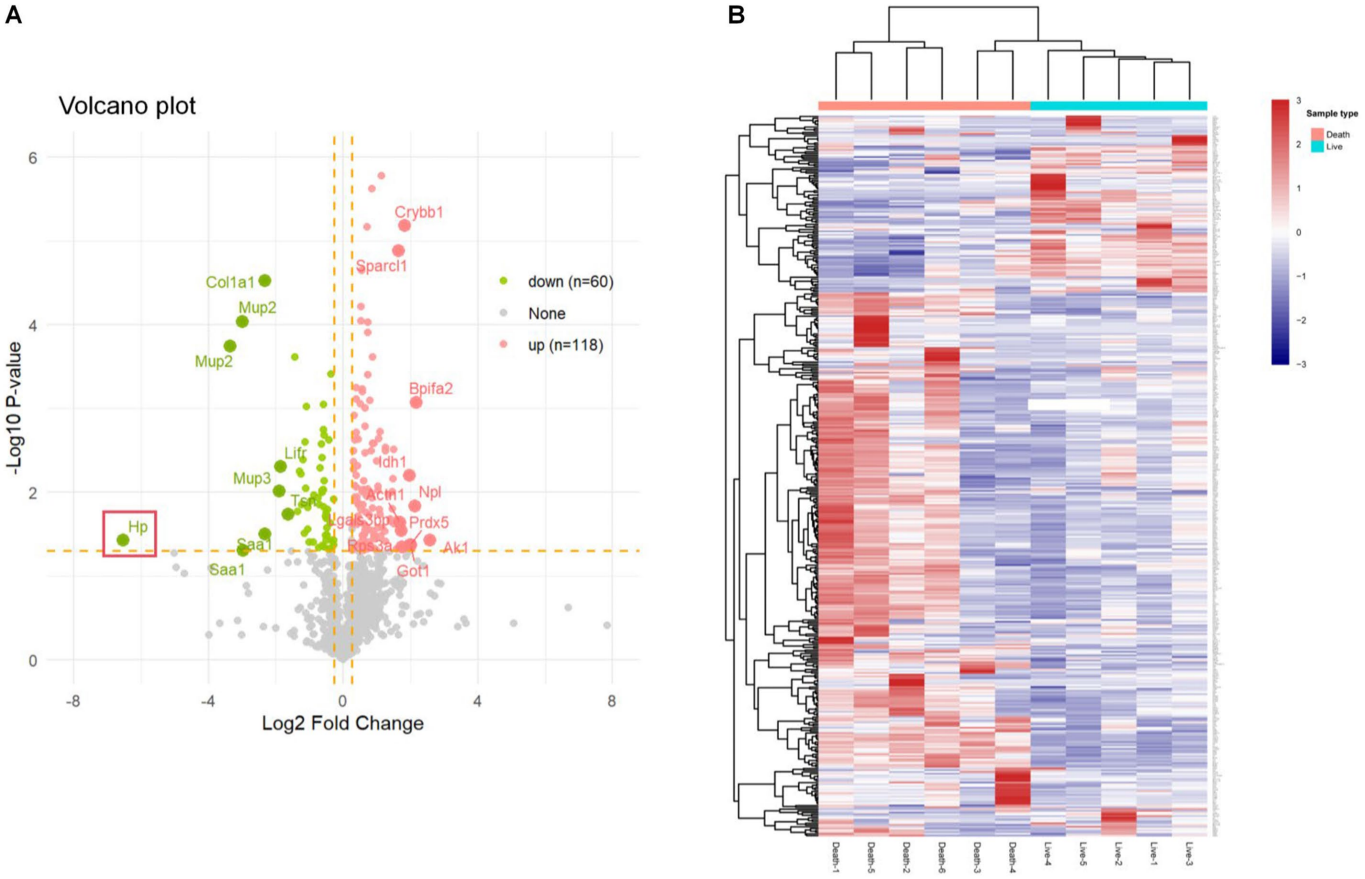
Differentially expressed proteins in survival group and non-survival group. **(A)** Volcano plot of differentially expressed proteins; the X axis represents protein difference (log2-transformed fold changes), and the Y axis represents the corresponding-log10-transformed *p* values. Red dots show significantly upregulated proteins, green dots indicate significantly downregulated proteins, and gray dots denote no significant proteins. **(B)** Hierarchical cluster analysis results of DEPs between the survival group and non-survival group. Higher red and blue intensities show higher degree of upregulation and downregulation, respectively.

In our DIA proteomic analysis, proteins were screened based on the criteria of *p* < 0.05 and FC ratio ≥ 1.20. Among the DEPs, 118 proteins exhibited significant upregulation (depicted as red dots) and 60 proteins showed significant downregulation (depicted as green dots) in the plasma of non-surviving DAH mice. The remaining proteins did not show significant differences (depicted as grey dots) (Figure 2A). The top 10 upregulated and downregulated DEPs are presented in Tables 1, 2, respectively. Notably, haptoglobin was the most downregulated in the non-survival group, which was highlighted with a gene tag in Figure 2A. Cluster analysis and heatmap data illustrated distinct variations in protein abundance between the survival and non-survival groups (Figure 2B).

**Table 1:** Top 10 down-regulated proteins.

Gene	Protein	*p* value	log2FC	Description
Hp	Q3UBS3	0.037499937	−6.5491337	Haptoglobin
Mup2	A2AKN9	0.000179634	−3.3645799	Major urinary protein 2
Mup2	P11589	9.15E-05	−2.9929619	Major urinary protein 2
Saa1	P05366	0.049622986	−2.9842841	Serum amyloid A-1 protein
Col1a1	P11087	2.97E-05	−2.333453	Collagen alpha-1(I) chain
Saa1	Q3UWC2	0.031536722	−2.3325892	Serum amyloid A protein
Mup3	P04939	0.009800692	−1.8980171	Major urinary protein 3
Lifr	P42703	0.004980746	−1.8695212	Leukemia inhibitory factor receptor
Tsn	Q545E6	0.018408899	−1.6504783	RNA-binding protein
Pi16	Q9ET66	0.000244587	−1.4354776	Peptidase inhibitor 16

**Table 2:** Top 10 upregulated proteins.

Gene	Protein	*p* value	log2FC	Description
Ak1	Q9R0Y5	0.037271072	2.55892336	Adenylate kinase isoenzyme 1
Bpifa2	B7ZCG3	0.000846973	2.15263058	BPI fold-containing family A member 2 (Fragment)
Npl	Q9DCJ9	0.014684684	2.1166835	N-acetylneuraminate lyase
Got1	F7ALS6	0.042934494	2.00878724	Aspartate aminotransferase, cytoplasmic (Fragment)
Prdx5	A0A494BAZ4	0.042607878	1.97175899	Peroxiredoxin (Fragment)
Idh1	O88844	0.006356419	1.96434272	Isocitrate dehydrogenase [NADP] cytoplasmic
Crybb1	E9PYP8	6.60E-06	1.82164784	Beta-crystallin B1 (Fragment)
Rps3a	P97351	0.045362118	1.7419547	40S ribosomal protein S3a
Lgals3bp	Q07797	0.028934812	1.72109971	Galectin-3-binding protein
Actn1	A1BN54	0.023062221	1.6843111	Alpha actinin 1a

**Figure 3:**
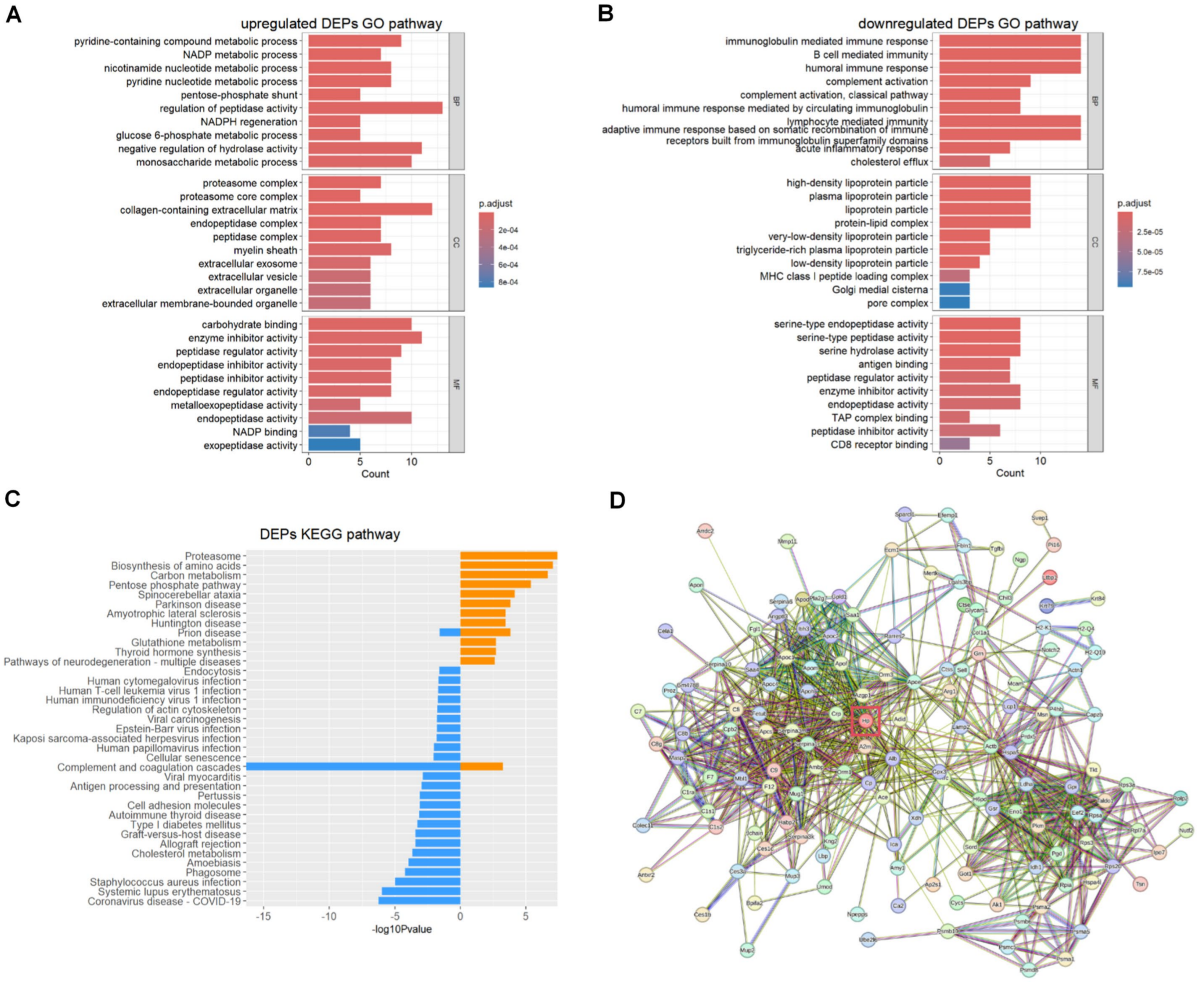
Upregulated DEP and downregulated DEP biological process analysis. **(A)** Upregulated DEP GO analysis. **(B)** Downregulated DEP GO analysis. **(C)** Upregulated (yellow) and downregulated (blue) DEP KEGG analysis. **(D)** Protein–protein interaction of vital DEPs.

Using the Gene Ontology (GO) database and Kyoto Encyclopedia of Genes and Genomes (KEGG) pathway database, we explored potential relationships and interactions between the upregulated and downregulated DEPs in the non-survival group of the DAH murine model. GO biological process (BP) analysis (Figures 3A,B) revealed that the upregulated DEPs are primarily involved in metabolic processes associated with the disease progression. In contrast, the downregulated DEPs are mainly associated with the immune response, including adaptive immune response, acute inflammatory response, and complement activation, particularly the classical pathway. Further cellular component (CC) analysis indicated that the upregulated DEPs are mainly derived from the proteasome complex and collagen-containing extracellular matrix. These proteins exhibit molecular functions (MF) such as carbohydrate binding and peptidase regulator activity. Conversely, the downregulated DEPs predominantly originate from lipoprotein particles and protein–lipid complexes, exhibiting molecular functions such as endopeptidase activity. KEGG pathway analysis (Figure 3C) showed that the upregulated DEPs are primarily involved in pathways related to the proteasome, biosynthesis of amino acids, and carbon metabolism. In contrast, the downregulated DEPs are mostly enriched in pathways related to complement and coagulation cascades, COVID-19, and systemic lupus erythematosus (SLE). As haptoglobin (Hp) is the most significantly downregulated DEP, protein–protein interaction (PPI) networks generated using the STRING database revealed the central role of Hp (as presented in Figure 3D) in DAH mice with poor outcomes.

### Reduction of haptoglobin in DAH mice and SLE-DAH patients

**Figure 4:**
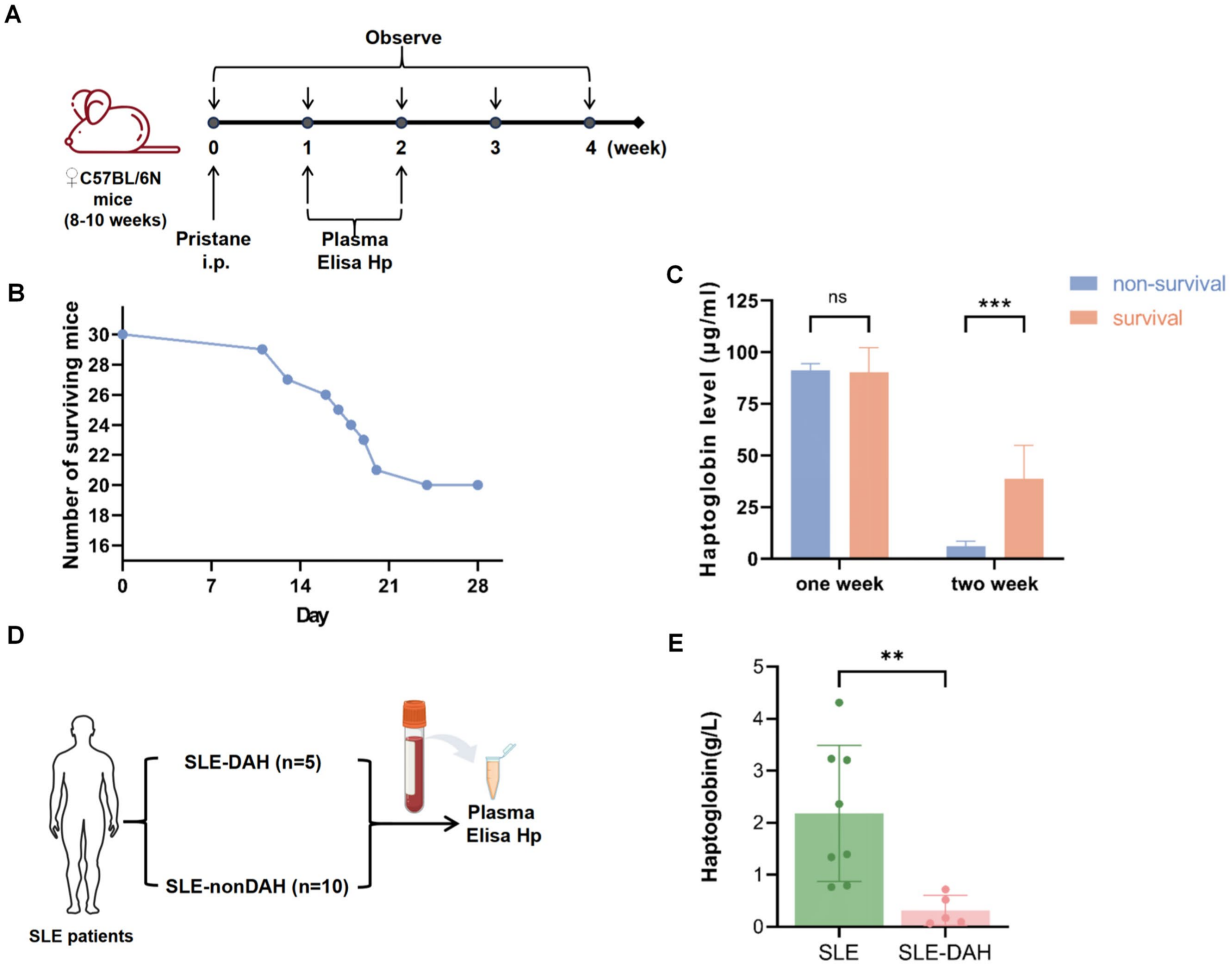
Validation of the haptoglobin descend in DAH. **(A)** Experimental setting for the DAH murine model (*n* = 30) with 500 μL pristane and observed for 4 weeks. **(B)** The number of survival and death of mice after injection pristane for 4 weeks. **(C)** Haptoglobin levels in mice plasma after pristane injection of 1 week and 2 weeks in survival group and non-survival group (values are the mean ± SD; *** = *p* < 0.001; unpaired t-test). **(D)** Experimental setting for SLE patients. **(E)** Haptoglobin levels in plasma from SLE with DAH patients compared with patients without SLE (values are the mean ± SD; ** = *p* < 0.01; unpaired *t*-test).

We induced a DAH model in C57BL/6 mice using pristane for 4 weeks and collected peripheral blood samples after 1 week and 2 weeks (Figure 4A). By the 28th day, 20 mice were alive and 10 had died (Figure 4B). The results showed that the levels of haptoglobin in the plasma of non-surviving mice were lower than those in the surviving group after 2 weeks of pristane treatment (Figure 4C). We also collected blood samples from 5 patients diagnosed with SLE and DAH and 8 patients diagnosed with SLE but without DAH to measure haptoglobin levels in the peripheral plasma (Figure 4D). The results indicated that haptoglobin levels were lower in patients with SLE-DAH compared to those with SLE without DAH (Figure 4E). These findings support decreased haptoglobin as an important factor associated with DAH, with more pronounced depletion correlating with a worse prognosis.

### The function of haptoglobin in pristane-induced DAH model

**Figure 5:**
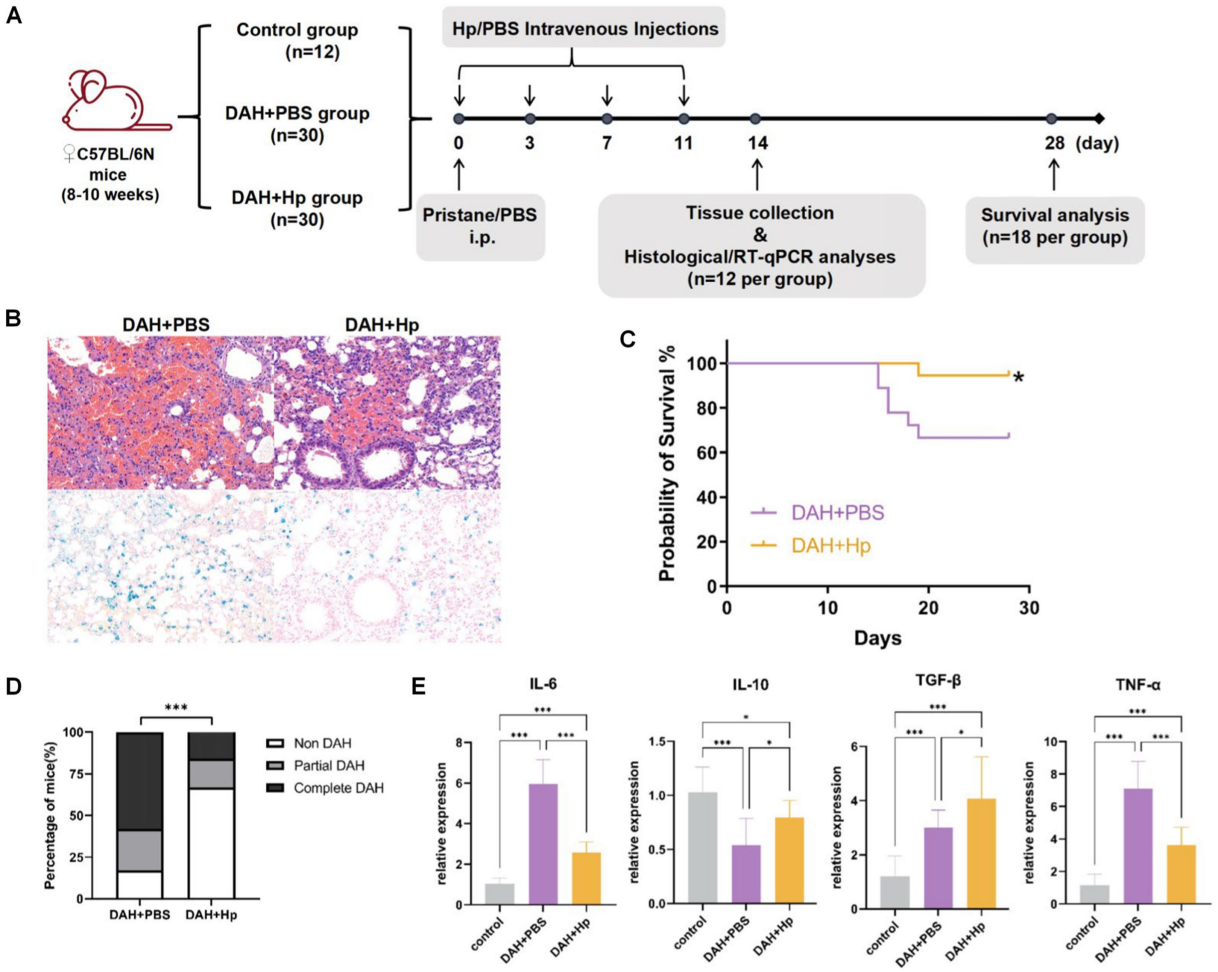
Validate the haptoglobin protective function in the DAH model. **(A)** Experimental setting for the *in vivo* tests; 2 weeks after pristane treatment, the prevalence of DAH was evaluated in histological and RT-qPCR analysis (*n* = 12 per group). **(B)** The 28-day survival curves of mice in DAH+Hp and DAH+PBS groups (*n* = 18) (*p < 0.05). **(C)** Representative micrograph images of H&E staining and Prussian blue staining lung sections in DAH+Hp and DAH+PBS groups (*n* = 12) at 2-week follow up (All images 200x). **(D)** Incidence of DAH on day 14 in the DAH and DAH+Hp groups (***p < 0.001, chi-square). **(E)** RT-qPCR analysis of cytokine mRNA relative expression level related to inflammation in the control, DAH, and DAH+Hp groups (*p < 0.05, ***p < 0.001; one-way ANOVA analysis).

To further validate the *in vivo* function of haptoglobin in the DAH murine model, we established Hp treatment on the DAH model (Figure 5A). The DAH+Hp group showed significantly decreased mortality on the 30th day compared with the DAH+PBS group (*p* < 0.05) (Figure 5B). Then, the lung tissue obtained at day 14 was analyzed, and H&E staining and Prussian blue staining suggested that hemorrhage and small-vessel vasculitis in the mouse lungs received remarkable alleviation after haptoglobin injection (Figure 5C). As shown in Figure 5D, lung pathology specimen from the haptoglobin-treated group demonstrated a better condition. Furthermore, RT-qPCR analysis of lung tissues from three groups of mice obtained on the 14th day revealed a reduction in inflammatory cytokines in the DAH model mice in the DAH+Hp group. The levels of inflammatory cytokines IL-6 and TNF-α were significantly decreased by the Hp treatment, while the levels of anti-inflammatory cytokines IL-10 and TGF-β were elevated after the Hp treatment (Figure 5E). These results suggest that the protective mechanism of haptoglobin in DAH may be related to the reduction in pro-inflammatory cytokines in the lung tissue. In conclusion, these findings indicate that Hp exerts a protective effect by reducing hemorrhage, inflammatory cell infiltration, and the production of inflammatory factors in the lungs, thereby preventing the occurrence of DAH.

## Discussion

Pristane-treated C57BL/6 mice serve as a classic model of SLE-DAH, primarily characterized by diffuse alveolar hemorrhage and mild kidney disease, unlike BALB/c mice, which do not exhibit lung injury (24). This model partially mimics the disease pathology observed in SLE-DAH patients, such as impaired clearance of apoptotic cells in the lung, presence of hemosiderin-laden macrophages, and deposition of immunoglobulins and complement (16). Furthermore, mounting evidence links DAH with poor prognosis and high mortality in SLE (2). Therefore, investigating key factors for alleviating DAH holds great clinical significance for improving prognosis and reducing mortality. In our study, we used the DIA technique to detect DEPs by collecting plasma samples from DAH murine models, followed by GO and KEGG pathway analysis. The findings were then validated in animal model interventions and SLE-DAH patients, offering new insights into the mechanism of DAH.

Our proteomic analysis identified 178 DEPs from DAH mice with different outcomes, of which 118 were upregulated and 60 were downregulated in the non-survival mice. The results of GO and KEGG analyses of all DEPs revealed the significant enrichment of the complement activation pathway and coagulation cascades. During the progression of the disease, the abnormal activation of the complement pathway could lead to sustained and exacerbated inflammatory responses, thereby exerting adverse effects on the organism and subsequently impacting the survival of the mice. Based on the upregulated DEP GO BP analysis, we observed that the non-survival group experienced more severe dysregulation of metabolic processes compared to the survival group after administering pristane. Conversely, the downregulation of immune responses in the non-survival group may represent a complex response of the body to the severe state, involving changes in multiple immune regulation and metabolic pathways. This may be due to immune exhaustion, where sustained inflammatory stimuli impair the function of key immune cells or signaling molecules, leading to apoptosis or necrosis and subsequent downregulation of the immune response. The imbalance of immune regulation potentially leads to the suppression of some immune response pathways, possibly contributing to the poor prognosis.

Interestingly, several similarities between COVID-19 and this disease were found in the KEGG pathway analysis. Autopsy findings of 21 COVID-19 patients indicated that respiratory failure, accompanied by infiltrated oriental alveolar injury and severe capillary congestion, was the primary cause of death (25). The involvement of the complement pathway, specifically the release of anaphylatoxins C3a and C5a through cleavage of C3 and C5 (26), as well as the coagulation cascade contributing to intravascular coagulation in the lung (27), has been implicated in the mechanism of COVID-19. Clinical trials have reported that tissue plasminogen activator (TPA) treatment could improve respiratory status in patients with severe COVID-19 (28). Additionally, the inhibition of fibrinolytic protease has been shown to reduce the imbalance between thrombosis and thrombotic cascade, thereby alleviating inflammation in the DAH murine model (29, 30). Several studies have also suggested that Hp is an anti-inflammatory acute phase protein, with its serum level associated with the transmission and severity of COVID-19 infection (31, 32). These results reveal the potential similarity in the pathogenesis of the two different diseases, SLE-DAH and COVID-19, providing an interesting direction for further study.

Our results showed that the expression of Hp was most significantly downregulated in the non-survival group of the DAH murine model. This was later validated in plasma samples from DAH mice in non-survival and survival groups by ELISA, showing a significant difference in Hp levels after 2 weeks of pristane treatment, suggesting that Hp depletion indicates a worse prognosis. Additionally, Hp depletion was also observed in SLE patients with DAH, indicating its clinical significance in evaluating DAH. To assess the therapeutic value of Hp, we found that the Hp-treated group had a significantly lower mortality rate than the untreated group at 28 days of follow-up. During 2 weeks, the degree of DAH was significantly reduced, and the expression of pro-inflammatory factors was decreased. These results supported the notion that Hp plays a crucial protective role by reducing hemorrhage severity, inflammatory cell infiltration, and inflammatory factor production in the lungs, preventing the occurrence of DAH. Thus, the depletion of Hp predicts a worse prognosis for DAH.

To the best of our knowledge, our study represents the initial exploration of proteomic analysis in a DAH murine model, providing valuable insights into the pathogenesis of DAH. More importantly, our research has demonstrated significant effects of the Hp treatment in improving survival rates and reducing pro-inflammatory cytokines in pristane-induced DAH, suggesting its therapeutic potential in diseases associated with DAH. However, our study has inherent limitations. First, although we have demonstrated the potential therapeutic effects of Hp on DAH, we have not yet explored the specific mechanisms *in vitro*. Some studies suggest that these mechanisms may involve CD163-mediated endocytosis in alveolar macrophages, which could reduce pro-inflammatory damage, oxidative stress, and the formation of neutrophil extracellular traps (NETs) (21, 33–35). This hypothesis, however, requires further in-depth investigation. Additionally, due to the limited availability of clinical samples, we have not been able to examine the clinical significance of Hp, including its correlation with the severity of DAH. Future research should aim to further investigate the mechanism by which Hp treats DAH, to explore more effective therapeutic strategies.

## Materials and methods

### Mice and experimental design

Female C57BL/6 mice aged 8–10 weeks, with an average weight of approximately 20 ± 2 g, were kept in the institutional animal facilities under controlled temperature, humidity, and light conditions and were given standard rodent feed. The animals were kept in the Animal Laboratory of Renji Hospital. All experimental operations and procedures were strictly in compliance with the regulations on experimental animals and animal ethics and were approved by the Animal Care and Use Committee of Renji Hospital, Shanghai Jiao Tong University School of Medicine. All mice that survived to the end point were euthanized with an intraperitoneal injection (i.p.) of an overdose of tribromoethanol (36).

Sixty female C57BL/6 mice received a single intraperitoneal injection (i.p.) of 0.5 mL pristane (Sigma) to induce the DAH murine model and were observed for 60 days, with their survival rate recorded. Throughout the observation period, approximately 0.2 mL of peripheral blood was collected from the posterior orbital venous plexus of surviving mice on days 0, 7, 14, 28, 42, and 60, respectively, and stored at −80°C for later use. Based on the survival status of the mice on day 60, the mice were divided into a survival group and a non-survival group. Peripheral blood samples were collected on day 14th from each group and were used for DIA analysis.

Thirty female C57BL/6 DAH murine models were then observed for 4 weeks, and their survival situation was counted. According to the survival situations of the mice on day 28, the mice were divided into survival group and non-survival group. During the observation period, approximately 0.2 mL of peripheral blood was taken from the posterior orbital venous plexus of each group on the 7th day and 14th day, respectively, for ELISA analysis of Hp.

Seventy-two C57BL/6 mice were randomly divided into three groups: (1) DAH group (*n* = 30): mice received a single intraperitoneal injection of 0.5 mL pristane on day 0, followed by tail vein injections of PBS (100 μL per mouse) on days 0, 3, 7, and 11; (2) DAH+Hp group (*n* = 30): mice received a single intraperitoneal injection of 0.5 mL pristane on day 0, followed by tail vein injections of a 2.5 mg/mL Hp (Sigma) suspension (100 μL per mouse) on days 0, 3, 7, and 11; and (3) control group (*n* = 12): mice received a single intraperitoneal injection of 0.5 mL PBS on day 0, followed by tail vein injections of PBS (100 μL per mouse) on days 0, 3, 7, and 11. Lung tissue and peripheral blood samples on day 14 of each group (*n* = 12 per group) were collected after euthanasia for *in vivo* validation experiments. The remaining 18 mice in each group were included in a 28-day survival analysis.

### DIA proteomics analysis

To perform the DIA proteomics analysis, plasma samples were first collected from non-survivors (*n* = 6) and survivors (*n* = 5). These samples were subjected to acetone precipitation to precipitate proteins and subsequently enriched using the ProteoMiner™ protein enrichment kit (Bio-Rad/1633007), to remove high-abundance proteins and isolate low-abundance proteins. The protein concentration of the samples was then determined using the Bradford protein quantification kit. Following this, the protein samples were digested with trypsin to produce peptides.

The digested peptide samples were injected into the EASY-nLCTM 1,200 UHPLC system (Thermo Fisher) coupled with an Orbitrap Q Exactive HF-X mass spectrometer (Thermo Fisher) operating in the data-independent acquisition (DIA) mode. For DIA acquisition, the mass-to-charge ratio (m/z) ranges from 350 to 1,500 using 36 scan windows. Finally, the raw mass spectrometry detection data were generated for further analysis.

Proteome Discoverer 2.2 (PD2.2, Thermo Fisher) software was used to search the raw data against the mouse database reviewed by UniProt (2020_7_2, 86,555 sequences) and was further filtered. After protein quantification and calculation, DEPs were selected: upregulated proteins were selected when FC ≥ 1.2 and *p* ≤ 0.05, while downregulated proteins were selected when FC ≤ 0.83 and *p* ≤ 0.05. The ‘clusterProfiler’ software package was used for Gene Ontology (GO) classification and Kyoto Encyclopedia of Genes and Genomes (KEGG) enrichment of DEPs (*p* < 0.05). The STRING database^1^ was used to analyze potential protein–protein interactions.

### Human peripheral blood sample origin and collection

Thirteen age- and sex-matched patients diagnosed as “SLE with DAH” or “SLE without DAH” were selected from Renji Hospital from January 2022 to December 2022. All the patients met the diagnostic criteria of SLE: according to the criteria of the American Rheumatology Association in 1997 (37), the patients were antibody-positive, anti-nuclear antibodies, and/or anti-double stranded DNA (anti-dsDNA) antibodies. Moreover, the Safety of Estrogens in Lupus Erythematous National Assessment (SELENA-SLEDAI) version of the systematic lupus erythematous disease activity index (SLEAI) with a score of ≥8 (range 0–105) is satisfied. Diagnostic criteria of DAH are as follows: a. pulmonary symptoms such as cough, hemoptysis, dyspnea, and hypoxemia; b. pulmonary infiltration appeared in imaging; c. a decrease in hemoglobin is greater than or equal to 15 g/L; d. bronchoalveolar lavage fluid (BALF) is bloody or hemosiderin cells can be observed (38). SLE-DAH patients can be diagnosed by meeting at least three of the four criteria. Meanwhile, all patients were excluded from the following diseases: severe abnormal coagulation diseases, acute pulmonary edema, pulmonary embolism, severe cardiac insufficiency, and conventional lung infections caused by bacteria, fungi, and viruses (6, 39). Serum was collected from 5 mL peripheral blood samples of 13 patients and stored at −80°C. The study was approved by the Ethics Committee of Ren Ji Hospital, Shanghai Jiao Tong University School of Medicine.

### Histopathological examination

The lung tissue of mice was perfused with 10% formalin solution and fixed overnight at room temperature. After paraffin embedding, 4 μm thick lung sections were taken, and the lung structure and inflammatory cell infiltration were analyzed by hematoxylin–eosin (H&E) and Prussian blue staining.

### ELISA analysis

The plasma of mice and patients was obtained from the collected peripheral blood. According to the manufacturer’s instructions, the content of Hp in plasma samples of mice and patients was determined, respectively, by ELISA Mouse Haptoglobin (Dy4409) and Human Haptoglobin (Dy8465) kit (R&D Systems). The OD value of each well at the wavelength of 450 nm was detected by a microplate analyzer, and the protein concentration was calculated according to the instructions and the protein standard curve.

### RT-qPCR

According to the manufacturer’s instructions, 2–3 steel balls and 1 mL of TRIZOL reagent were added to the lung tissue obtained on day 14 to extract the total RNA. Using Evo M-MLV reverse transcription reagent to reverse transcribe RNA, set the PCR program to 37°C for 15 min, 85°C for 5 s, and 4°C ∞ to obtain cDNA. Real-time fluorescence quantitative PCR (RT-qPCR) was performed using SYBR Green Master Mix and Thermo Fisher Scientific. The amplification conditions were 95°C 30 s, followed by 40 cycles, 5 s at 95°C and 30 s at 60°C. Then, the melting curve was analyzed and verified, and the relative expression was calculated by the 2–ΔΔCt method. The primer sequences were summarized: IL-6, CTGCAAGAGACTTCCATCCAG (F 5′ to 3′), AGTGGTATAGACAGGTCTGTTGG (R 5′ to 3′); IL-10, TGAATTCCCTGGGTGAGAAGC (F 5′ to 3′), CACCTTGGTCTTGGAGCTTATT (R 5′ to 3′); TNF-α, CCCTCCAGAAAAGACACCATG (F 5′ to 3′), GCCACAAGCAGGAATGAGAAG (R 5′ to 3′); TGF-β, GCAACAATTCCTGGCGTTA (F 5′ to 3′), TTCCGTCTCCTTGGTTCAG (R 5′ to 3′); Actin-β, AAAACTGGAACGGTGAAGGC (F 5′ to 3′), GTCCTCAGCCACATTTGTAGA (R 5′ to 3′).

### Statistical analysis

Data were analyzed and plotted with GraphPad Prism (version 9.02) and R studio (version 4.3.0). Mann–Whitney U-test or t-test was used for the comparison of two groups according to the normality of the data. One-way analysis of variance (ANOVA) was used for more than two independent groups. All analyses were performed at least three times, and representative experimental results were provided. *p*-values of less than 0.05 were considered statistically significant.

## Data availability statement

The data presented in the study are deposited in the NODE repository by passing the accession number OEZ017560 into the search box or through the URL: https://www.biosino.org/node/analysis/detail/OEZ017560.

## Ethics statement

The studies involving humans were approved by the Ethics Committee of Ren Ji Hospital, Shanghai Jiao Tong University School of Medicine. The studies were conducted in accordance with the local legislation and institutional requirements. The participants provided their written informed consent to participate in this study. The animal study was approved by the Animal Care and Use Committee of Ren Ji Hospital, Shanghai Jiao Tong University School of Medicine. The study was conducted in accordance with the local legislation and institutional requirements.

## Author contributions

NY: Conceptualization, Data curation, Formal analysis, Investigation, Methodology, Software, Validation, Visualization, Writing – original draft, Writing – review & editing. CS: Conceptualization, Data curation, Formal analysis, Investigation, Methodology, Software, Validation, Visualization, Writing – original draft, Writing – review & editing. YZ: Investigation, Methodology, Software, Validation, Visualization, Writing – original draft, Writing – review & editing. XZ: Software, Supervision, Validation, Visualization, Writing – original draft, Writing – review & editing. NX: Software, Supervision, Validation, Visualization, Writing – original draft, Writing – review & editing. QG: Conceptualization, Funding acquisition, Methodology, Project administration, Resources, Supervision, Writing – original draft, Writing – review & editing.

## Footnotes

^1^ http://www.string-db.org
