# Supplementary material for: Quantitative proteomic analysis and replacement therapy identifies haptoglobin as a therapeutic target in a murine model of SLE-associated diffuse alveolar hemorrhage
Source: Front Vet Sci. 2024 Aug 12;11:1431738. doi: 10.3389/fvets.2024.1431738 (PMC11345213; doi:10.3389/fvets.2024.1431738)
Supplement: Supplementary file 1 [file Data_Sheet_1.DOCX]

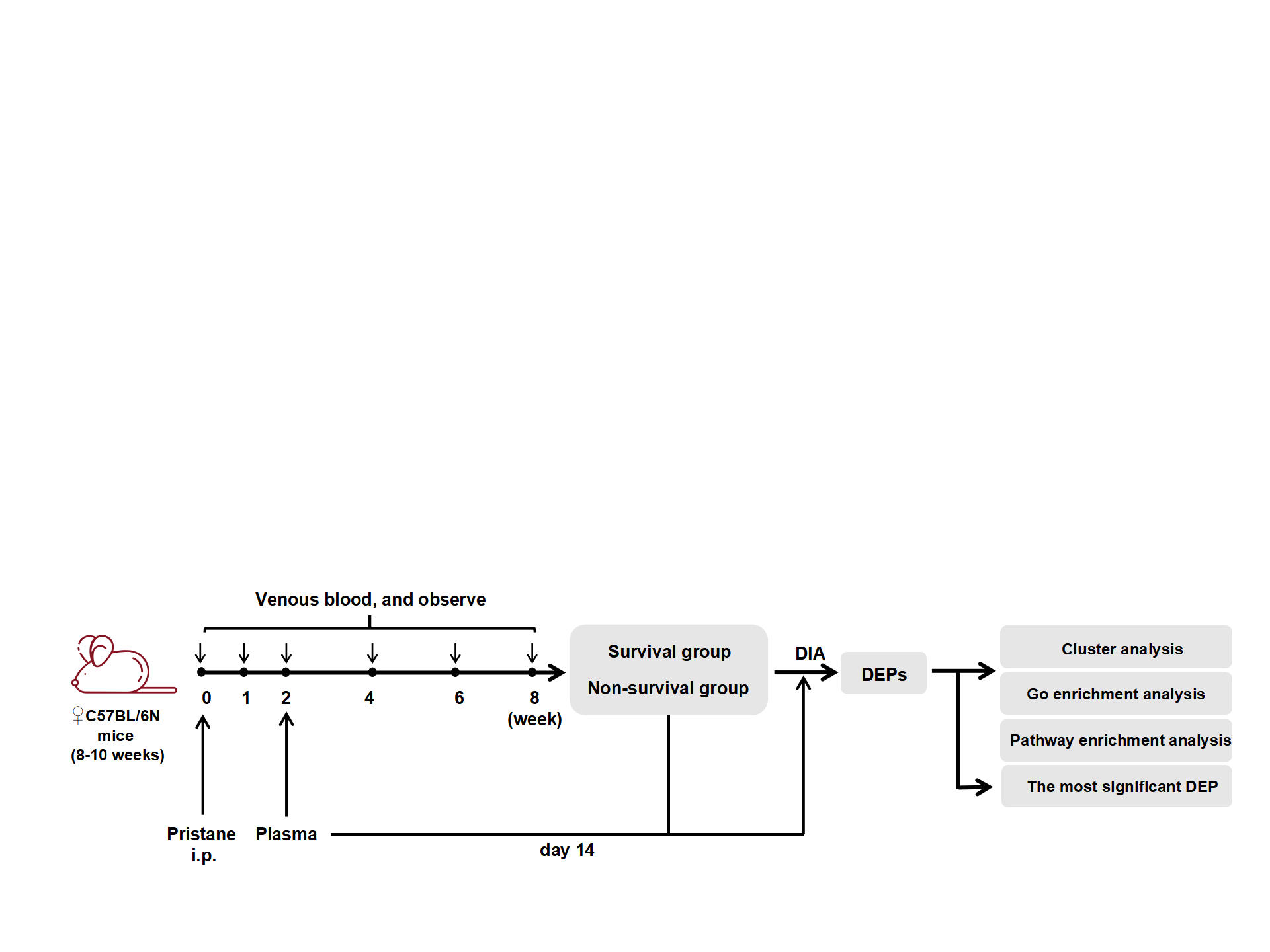


**Supplementary Figure S1. General workflow and experimental design**

1. week-old female C57BL/6 mice were treated with i.p. with 0.5 ml pristane on day-0 and mice were sacrificed for analysis after pristane injection 2 weeks. All the mice were obtained 0.2 ml of peripheral blood on day 0, 7, 14, 28, 42, and 60 respectively. According to the survival situation of mice on day-60, peripheral blood on day-14 were used for DIA proteomic analysis, which included Cluster analysis, GO enrichment analysis, Pathway enrichment analysis and finding out the most significant DEP.

**
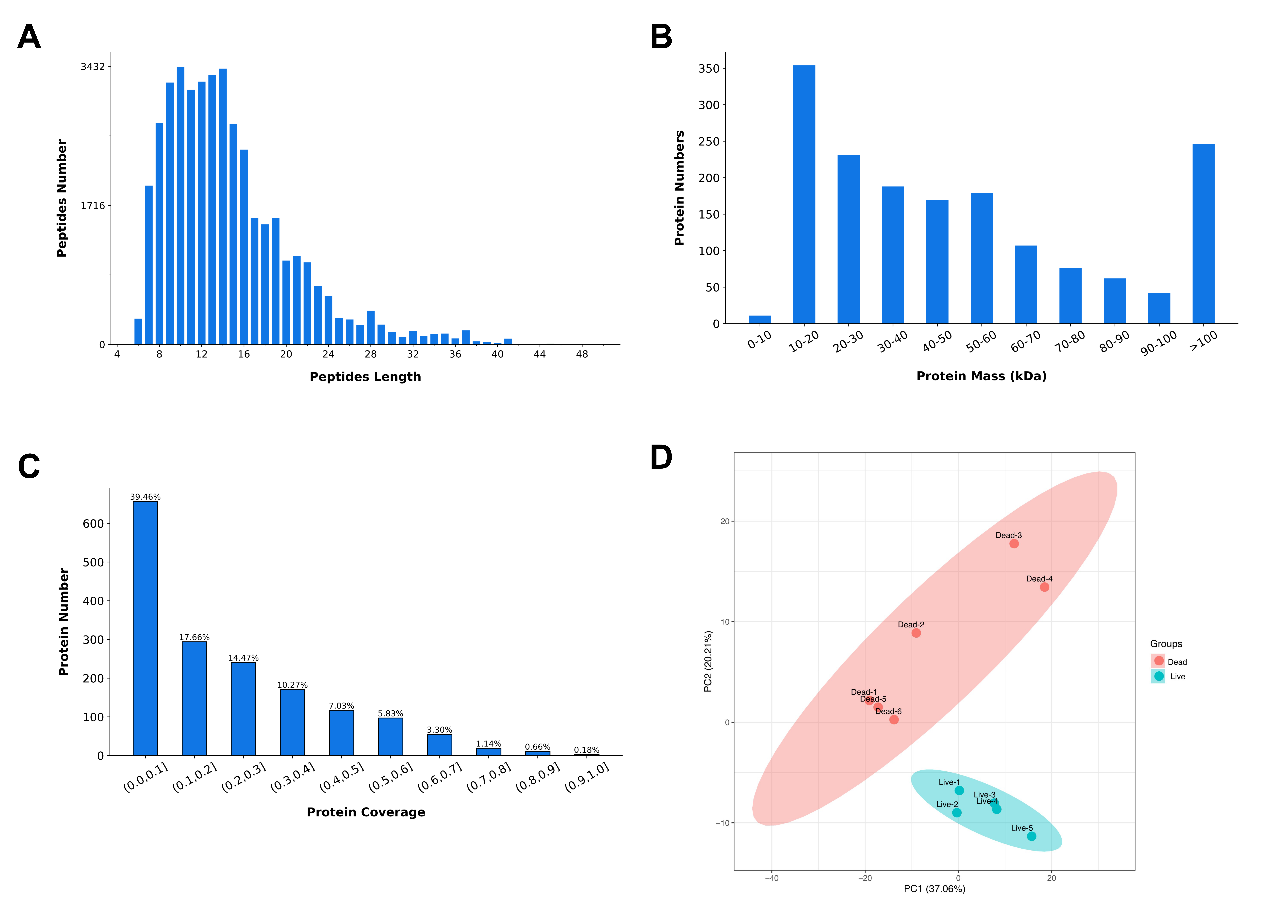
 Supplementary Figure S2 General results in proteomics.**

A. Length distribution of the peptides identified by mass spectrometry.

B. Protein weight distribution of the peptides identified by mass spectrometry.

C. Relationship between protein molecular weight and coverage.

D. Principal component analysis (PCA) between survival group and non-survival group
